# “Voices of Fear and Safety” Women’s ambivalence towards breast cancer and breast health: a qualitative study from Jordan

**DOI:** 10.1186/1472-6874-12-21

**Published:** 2012-07-26

**Authors:** Hana Taha, Raeda Al-Qutob, Lennarth Nyström, Rolf Wahlström, Vanja Berggren

**Affiliations:** 1 Division of Global Health (IHCAR), Department of Public Health Sciences, Karolinska Institutet, Stockholm, Sweden; 2 Jordan Breast Cancer Program, Amman, Jordan; 3 King Hussein Cancer Foundation, Amman, Jordan; 4 Women and Child Health Division, Department of Family and Community Medicine, University of Jordan, Amman, Jordan; 5 Higher Population Council, Amman, Jordan; 6 Division of Epidemiology and Global Health, Department of Public Health and Clinical Medicine, Umeå University, Umeå, Sweden; 7 Family Medicine and Clinical Epidemiology, Department of Public Health and Care Sciences, Uppsala University, Uppsala, Sweden

## Abstract

**Background:**

Breast cancer is the leading cause of cancer mortality among Jordanian women. Breast malignancies are detected at late stages as a result of deferred breast health-seeking behaviour. The aim of this study was to explore Jordanian women’s views and perceptions about breast cancer and breast health.

**Methods:**

We performed an explorative qualitative study with purposive sampling. Ten focus groups were conducted consisting of 64 women (aged 20 to 65 years) with no previous history and no symptoms of breast cancer from four governorates in Jordan. The transcribed data was analysed using latent content analysis.

**Results:**

Three themes were constructed from the group discussions: a) Ambivalence in prioritizing own health; b) Feeling fear of breast cancer; and c) Feeling safe from breast cancer. The first theme was seen in women’s prioritizing children and family needs and in their experiencing family and social support towards seeking breast health care. The second theme was building on women’s perception of breast cancer as an incurable disease associated with suffering and death, their fear of the risk of diminished femininity, husband’s rejection and social stigmatization, adding to their apprehensions about breast health examinations. The third theme emerged from the women’s perceiving themselves as not being in the risk zone for breast cancer and in their accepting breast cancer as a test from God. In contrast, women also experienced comfort in acquiring breast health knowledge that soothed their fears and motivated them to seek early detection examinations.

**Conclusions:**

Women’s ambivalence in prioritizing their own health and feelings of fear and safety could be better addressed by designing breast health interventions that emphasize the good prognosis for breast cancer when detected early, involve breast cancer survivors in breast health awareness campaigns and catalyse family support to encourage women to seek breast health care.

## Background

Breast cancer is the leading cause of cancer related mortality among women worldwide; it constitutes 23% of the total new cancer cases and 14% of the cancer deaths [1]. Early detection of breast cancer makes the treatment more effective which leads to better health outcomes and higher survival rates. The 5-year survival rate reaches 93 and 88% when breast cancer is detected in its earliest stages 0 and I respectively, compared to 15% in stage IV [2].

Breast cancer is the most common cancer in Jordan, constituting 20% of the total cancer cases and 22% of the cancer deaths. The age-standardized incidence rate of breast cancer increased from 29/100 000 in 1996 to 50/100 000 in 2008. Breast cancer comprises 37% of all female cancer cases in Jordan and the highest incidence is seen in women aged 40–49 years [3]. In 2006, 70% of the breast cancer cases in Jordan were diagnosed at advanced stages (Stage III-IV). Hence, the Jordan Breast Cancer Program (JBCP) was established in 2007 as a national initiative, led by the King Hussein Cancer Foundation (KHCF) to create public awareness and to ensure availability and accessibility to quality screening services in Jordan [4].

By 2008, JBCP was able to mobilize the Ministry of Health and create partnerships with the major stakeholders in Jordan’s health sector to build screening services and raise public awareness while institutionalizing policy. This has helped to bring about a significant reduction in late-stage diagnosis of breast cancer in Jordan. Statistics from the King Hussein Cancer Center (KHCC), which administers more than two-thirds of the Kingdom’s cancer cases, have shown a 50% reduction in the diagnosis of stage III and IV breast cancer cases [3,4].

There is still a gap of knowledge about the explanatory factors for the delayed presentation of breast cancer in the Middle East. A systematic review by Alhurishi et al. (2011) found six studies on the explanatory factors for the delayed presentation of breast cancer in the Middle East and all of them employed quantitative methods [5]. Older age and lower educational level were found to have strong effects in explaining late presentation. Having no family history of breast cancer was found to have moderate effect on breast cancer late presentation. There is a need for qualitative research to obtain a deeper understanding of the problem and to provide data for designing breast health promotion strategies that are culturally sensitive to Jordan. Thus, this study aimed to explore Jordanian women’s views and perceptions about breast cancer and breast health. The findings will be used for designing breast health promotion strategies that are culturally sensitive to Jordan.

## Methods

### Study setting

Jordan is a lower middle income country with a population of six million (49% females). Eight out of ten (83%) live in cities and the rest in rural areas and desert. In 2009, the average GDP per capita was 4 196 US $. All children receive ten years of compulsory basic education which is free of charge [6].

This study was conducted in four governorates; Amman, Irbid, Karak and Balqa. These four governorates constitute 70% of the total population and demonstrate the socio-cultural texture of the Jordanian society. Amman has a total population of 2.4 million (94% urban dwellers). There are clear socio-economic disparities between Amman’s western and eastern parts. West Amman is the affluent side of the city, while East Amman is the underprivileged side of the city. People in the respective parts of the city have different lifestyles, experiences, beliefs, and perceptions [7-9].

Irbid is located in the north of Jordan with a population of 1.1 million (83% urban and 17% rural), Karak is located in the south of Jordan with a population of 238 000 (35% urban and 65 % rural), and Balqa has a population of 410 000 (72% urban and 28 % rural) and is situated close to Amman [9]. Primary health care services in Jordan are subsidized by the Ministry of Health (MoH) and well accessible. There is a wide coverage nationwide; the estimated average travel time to reach the nearest health centre is 30 minutes, and the accessibility level is approximately 97% [10].

Although the benefits of breast self-examination (BSE) had not been confirmed indisputably in the literature [11,12] several studies have indicated that women who regularly practice BSE initially present with smaller tumours that less frequently involve the axillary lymph nodes [13-15]. Hence, the Jordan national breast health guidelines promote breast health awareness to all Jordanian women including a recommendation that women should start practicing monthly BSE from the age of 20 years [16]. This is consistent with the recommendations of the Breast Health Global Initiative (BHGI) for limited resources countries [17,18]. Studies have shown that using clinical breast examination (CBE) and mammography screening for early detection of breast cancer lead to down-staging at the onset of diagnosis and improved odds of survival [19]. In Jordan, CBE is recommended once every 1–3 years in the age group 20–39 years and annually in women aged 40 and above. Mammography is recommended once every 1–2 years starting from age 40 years and above [16].

Recently the MoH has passed a new law soon to be incorporated into the civil health insurance system. The law stipulates that all Jordanian women are entitled to free early detection examinations for breast cancer (CBE and mammography) which will be available at any of the public sector hospitals and health centers [20]. Based on the latest unpublished data from JBCP’s operations department about the mammography screening services in Jordan, on May 16th, 2012 there were 67 functional mammography units in Jordan, three of which were digital. They are unevenly distributed with higher coverage in urban areas; 28 of them are in the public sector, 31 in the private sector, 2 in the Royal Medical Services (RMS), 2 in KHCC and 4 in university hospitals. The mammography units in KHCC and RMS are extensively used, while those in the private and the public sector are underutilized.

### Study design

In this study we chose a qualitative approach to get a deeper understanding of the women’s experiences. We conducted focus group discussions (FGDs) to encourage the group dynamics and to generate collective experiences, views and perceptions about breast cancer and breast health [21].

### Study population

To maximize information richness, the participants in this study were selected purposively among woman aged 20 to 65 years with no previous history and no symptoms of breast cancer [21-23]. They displayed different attributes with regard to site of residence, social group, age and educational level. In total, 64 women with a median age of 38 years participated in ten FGDs, 5–8 participants in each FGD, in the four governorates, Amman, Balqa, Irbid and Karak. They were recruited through women’s nongovernmental organizations (NGOs) and from the clients of primary health care centers close to their homes.

Thirty-five of the women were from urban areas and 29 from rural areas; 39 of the women were married, 19 were single, three were divorced and three were widows; 36 women were 20 to 39 years old and 28 women were aged 40 to 65 years; 35 women were housewives, 23 were salaried employees, five were retired and one was a student; 40 women had a monthly income of less than 700 USD, 13 women had an income between 700 and 1400 USD, one woman had an income above 1400 USD, and ten women did not disclose their income; 12 women had primary education, 18 had finished high school, eight women had a precollege diploma, 23 had a bachelor’s degree and three had completed postgraduate studies.

### Data collection

The research team developed a guide for the FGDs based on a review of the literature. Box 1 shows the FGD guide that included open-ended and appropriate probing questions to encourage spontaneous dialogue among women about their perceptions of breast cancer and their views on early detection examinations. The principal investigator (PI) moderated two pilot FGDs in Arabic with 20–65 years old Jordanian women, after which the FGD guide was revised to facilitate discussion. We also decided to split the participants by age (20–39 years and 40–65 years) to overcome the shyness of the younger participants.

The FGDs took place in quiet rooms in a nearby women´s NGO or health center. In all the FDGs, the venues had comfortable round table organization and all the women had eye contact with each other throughout. All the FGDs were moderated by the PI in Arabic. Each lasted about 50–60 minutes. The FGDs were all audio-taped and an Arabic speaking research assistant attended to observe and take notes. The tape recorded data from all the FGDs, including the pilot ones, were transcribed in Arabic and thereafter half of them were translated to English for analysis by the English speaking co-researchers. Based on the flow of the information while the research was ongoing we stopped at ten FGDs when saturation and information redundancy occurred [24].

### Data analysis

Data from the FGDs was read by the PI and the co-researchers and analysis was conducted using latent content analysis [25]. The PI condensed the Arabic text into meaning units followed by English coding and categorization. The coding and categorization of the data was validated by the co researchers. Thereafter the PI and the co researchers clustered the categories into emerging themes. Triangulation of researchers was used to enhance the trustworthiness of the findings [26].

### Ethical considerations

The ethical clearance for this study was issued from the Jordan Ministry of Health Research Ethics Committee in 2009. The confidentiality and autonomy of the participants was insured. They were informed of the purpose of the study, the voluntary nature of their participation, and their right to access findings. Informed consent was sought from all participants.

## Results

Common patterns embracing the women’s views and perceptions about breast cancer and breast health were discussed in all the FGDs. The following description of themes was developed: a) Ambivalence in prioritizing one’s own health; b) Feeling fear of breast cancer, and c) Feeling safe from breast cancer. The first theme was seen in women’s prioritizing children and family needs and in their experiencing family and social support towards seeking breast health care. The second theme was building on women’s perception of breast cancer as an incurable disease associated with suffering and death, their fear of the risk of diminished femininity, husband’s rejection and social stigmatization, adding to their apprehensions about breast health examinations. The third theme emerged from the women’s perceiving themselves as not being in the risk zone for breast cancer and in their accepting breast cancer as a test from God. In contrast, women also experienced comfort in acquiring breast health knowledge that soothed their fears and motivated them to seek early detection examinations.

All the themes and categories are listed in Box 2. The themes are written in bold and the categories in bold-italic.

### Ambivalence in prioritizing own health

On one hand, women shared the experience that they prioritize children and family needs, at the cost of their own health, while, on the other hand, they told about receiving family and social support to prioritize their own health and seek breast health care.

#### Children and family come first

Giving priority to children and family above their own health was discussed. Women claimed that if there were enough resources they would take care of their own health, however, when there was limited money, women prioritized their children’s needs.

"*“If I have money allocated for my health, then my son needs money or my daughter wanted a dress, I would put their requests first and leave my own needs last” (4, 1)*"

This did not appear as prevalent in the FGDs with women from more affluent areas. They prioritized their children and family without neglecting their own health. Those women told about their own healthy practices that included diet, sports and seeking periodic screening for breast cancer.

"*“I do my chores but I try to take care of myself too, I don’t forget myself, because we usually pamper our children and forget ourselves” (6, 7)*"

In all the FGDs, women perceived their own health value from the perspective of being in charge of taking care of the family, and they mentioned that this was also the perception of their husbands.

"*“My health is important, because if something bad happens to me, my whole family will be lost, because the mother is the nerve of life” (4, 4)*"

#### *Family and social support towards seeking breast health care*

In all the FGDs, family and social support appeared to be a motivator that enabled women to overcome their ambivalence towards seeking breast health care. The women experienced and appreciated receiving encouragement from their husbands or their mothers to practice breast health care. They told about older daughters and sons booking the appointment and escorting them to the mammography unit. They also mentioned being reminded by a sister to practice BSE or being accompanied by a neighbour or a friend to go for CBE.

"*“My family considers my health first, but for me; my health is one of my priorities but not the first” (4, 7)*"

In all the FGDs except two, women commented that they did not feel they needed to ask for permission before seeking breast health care but they informed or consulted or were accompanied by the husband if married or the mother if single. The FGDs in which women felt that they needed the husband’s permission prior to seeking breast health care were from less privileged areas.

"*“I just tell him I am going to the doctor, he is my husband he has to know, but I don’t ask for his permission” (4, 8)*"

### Feeling fear of breast cancer

The second theme is built on four categories: a) perception of breast cancer as an incurable disease associated with suffering and death; b) fear of the risk of diminished femininity and husband’s rejection; c) fear of social stigmatization of the disease, and d) apprehensions about breast health examinations.

#### *An incurable disease associated with suffering and death*

In all the FGDs women perceived breast cancer as a source of suffering for the woman and her loved ones followed by death. Women questioned if there really is a possibility to be cured, telling about witnessing relatives or friends who had suffered this vicious disease (in Arabic*: khabeeth*). There were women who explained that even if a woman gets cured for a few years, the breast cancer will come back and kill her.

"*“Breast cancer means body disfigurement, suffering, family life disruption and death” (3, 1)*"

"*“My aunt, they removed her breast and she received chemo therapy and radiotherapy treatment, she was cured of it for 8 years, but she used to take medicine regularly every month to prevent the disease from spreading, but 8 years later it spread to her lung. She did not live long.” (4, 2)*"

However, in all the FGDs there were also stories about possible good prognosis of breast cancer when detected early.

"*“My colleague, they discovered her breast cancer in early stages, she was healed after receiving chemotherapy; without a mastectomy” (9, 7)*"

#### *Fear of the risk of diminished femininity and husband’s rejection*

In all the FGDs, women associated breast cancer with fear of a distorted body image and loss of femininity because it inflicts a body organ that symbolizes femininity and motherhood.

"*“A woman who gets breast cancer will be devastated; since losing her breasts means that she is finished as a woman and as a mother” (2, 2)*"

"*“We, women, care about beauty, and the breast is part of a woman's beauty that she needs to show her husband, isn’t it true? So her feeling of inferiority remains regardless of how well her husband deals with her, whether normally or with pity, or helps her or supports her psychological condition, this remains inside us” (9, 2)*"

It was a common perception that young women hit by breast cancer suffer more than older ones. The women reasoned that older women have grown-up children who would take care of them, while the younger women’s children are still too young and thus the younger woman will be more vulnerable if the husband rejects her.

"*“I know a young woman who had breast cancer; her husband married her best friend, Poor woman, her children are still young and can’t take care of her” (6, 2)*"

The women were of the opinion that there are few men who would stand by the wife if she had breast cancer. In all the FGDs, women had observed that men whose wives had been stricken by breast cancer had started looking for other women. They expressed that women in general are repressed in the society and considered by men as dolls.

"*“I know a woman who had breast cancer her husband rejected her and married another woman because she lost her femininity” (3, 8)*"

"*“In our society a woman is manipulated as a toy, a man whose wife gets inflicted with breast cancer, this hits his masculinity and usually immediately his eyes starts wandering after other women looking for a replacement” (6, 4)*"

"*“A man hates having a sick wife, he prefers that his wife stays healthy and strong, my neighbour had cancer, her husband and daughters felt sorry for her, however after a while her husband started looking for a new bride” (8, 3)*"

On the other hand, in some FGDs, women talked about husbands that supported the wife when she was inflicted with breast cancer.

"*“She had chemotherapy and as a result she became bold, her four sons along with their father cut their hair and became bold in solidarity with their mother” (6, 6)*"

In one FGD women talked about breast cancer being contagious and narrated about husbands rejecting their wives after they had been diagnosed with cancer because they were afraid they might catch the illness.

"*“These are viruses or bacteria that start eating the breast and continue to eat the whole body leading to death at the end” (4, 2)*"

"*“The husband said that this is a virus, a small organism inside the body which eats from the body, it would be possible that it can be transferred to him and live upon him too” (4, 7)*"

#### *Fear of social stigmatization*

Women in all FGDs told that breast cancer is a taboo subject in Jordan. The women explained that the word cancer by itself is a source of fear that is overstated by the society, which leads to it being referred to in people’s conversations as “that disease”. Women experienced that some women try to hide their illness because of fear of being socially stigmatized.

"*“A woman inflicted with breast cancer in our society hides having that illness, because breast is a sensitive issue for a woman and because that illness is considered to be vicious” (9, 4)*"

"*“Even she herself feels insecure after she has her breast removed, for example if you look at her and talk to her, she thinks that you are looking at the side where her breast was removed” (4, 8)*"

In all the FGDs women told that having a mother who had breast cancer might hinder the marriage of her daughters.

"*“When some people hear about a mother affected by breast cancer, they think that her daughter is going to be affected by the same disease due to heredity” (6, 2)*"

#### *Apprehensions about breast health examinations*

Women in all the FGDs discussed fear as a barrier that stopped them from practicing breast health examinations. Women told about avoiding touching their breasts or going for CBE or mammography because they feared finding a lump. Some women expressed that even if they had cancer, they did not want to know.

"*”I wish if that happened to me, God forbid, I wouldn't know and die without knowing about it” (5, 1)*"

On the other hand, in all the FGDs there were women who perceived that they are at higher risk of breast cancer due to having a personal or a family history of breast lumps or being childless or never having breastfed their children. These women had fear from breast cancer that outweighed their concerns towards screening. They told that they practice breast health examinations to be able to detect the disease at its earliest stages.

"*“I am scared, because I had a benign lump before and I did the surgery, now I do self-exam every month to be on the safe side.” (3, 2)*"

In some FGDs the women perceived mammography examination as painful and harmful. The women explained that such worries about possible harmful effects of x-rays were confirmed by their physicians.

"*“I asked the doctor whether I should do a mammogram test because it is easier and can show everything, she told me not to, and that I should first do physical manual examination, and that she does not advise me to do mammogram examination because the x-rays themselves affect the body negatively” (4, 2).*"

In all the FGDs some women expressed feeling uncomfortable and shy about having their CBE done by a male doctor.

"*”I have wanted to do it a long time ago but I have not found a female doctor, because it is impossible for me to visit a male doctor” (7, 2)*"

Shyness was also discussed as a barrier that stopped unmarried women from seeking breast health care. They commented that they felt embarrassed to talk about breast cancer or to seek breast health examinations because they were still unmarried.

### Feeling safe from breast cancer

The third theme emerged from the women’s perceiving themselves as not being in the risk zone for breast cancer and in their accepting breast cancer as a test from God. In contrast, women also experienced comfort in acquiring breast health knowledge that soothed their fears and motivated them to seek early detection examinations.

#### *Perceiving themselves as not being in the risk zone for breast cancer*

In all the focus groups women explained that they felt safe from breast cancer and did not seek CBE or mammography screening because they did not feel any symptoms or due to their doing BSE at home and not noticing any abnormal changes in the breasts.

"*“If, by the self-exam, something is found, I would go, but if nothing is wrong, why should I go to get a clinical examination” (9, 5)*"

In all the FGDs there were women who felt safe because they had breastfed their children.

"*“I don’t feel fear, I guess all is the result of God's will and moreover I breastfed so I hope all will be fine” (3, 8)*"

Women also expressed that they felt safe when the results of their first CBE or mammography screening were negative and because of that they did not feel a need to go for periodic examinations.

"*“I only did clinical examination and mammography once in my whole life, I had pain in my chest, and my examination results came normal, after that I didn’t feel that I need to go for periodic tests” (4, 2)*"

#### *Accepting breast cancer as a test from God*

The name of God was present in all the FGDs. In some FGDs women expressed that breast cancer is a test of human patience by God. They explained that they feel that breast examinations are not necessary since the issues of illness, life and death should rather be left to Allah Almighty. Whenever anyone mentioned this it was left without being questioned and it put a lid on the discussion.

"*“Last year my doctor referred me to mammography but I agreed with my husband not to do it, if God wanted to test me with such illness, then I accept God’s will, but I will not continue checking myself” (1, 1)*"

For the women who took this perspective, breast cancer was perceived as a plight from Allah and if a woman is destined to have cancer, no matter all her precautions, she will be inflicted.

"*“Glory be to God, it is a test from Allah, He wants to see if one can be patient or not” (4, 8)*"

At the same time as there were FGDs in which women expressed being tested by God, women in all the FGDs told that God created a cure for every illness.

"*“God created a cure for every illness and breast cancer does not mean the end of life” (2, 2)*"

#### *Comfort in acquiring breast health knowledge and skills*

In all the FGDs, women talked about seeing or hearing about breast cancer and breast health examinations on TV, radio, billboards, doctor’s clinics and newspapers. In addition, they talked about attending lectures on breast cancer at nearby NGOs or learning about how to do BSE from the physician in the maternity and child health care centres. The participants also talked about home visits by outreach workers to educate them about breast cancer. They expressed that their fears were soothed following to acquiring breast health knowledge and skills and this encouraged them to practice breast health examinations.

"*“I attended a lecture two years ago performed by a female doctor and I was encouraged to have my breasts examined” (6, 3)*"

In all the FGDs women experienced forgetfulness due to having many chores that keep them busy and distract them from practising SBE, seeking CBE or mammography. The women appreciated being reminded by the media and through other breast health promotion activities. It was commented that it would be valuable if women who actually had survived breast cancer participated in the breast health promotion activities.

"*“She should be a woman who has been cured after detecting her breast cancer early and receiving treatment for it, this would provide me with hope, as I would prefer to die and be buried in one piece than being cut and sold by kilo” (4, 7).*"

## Discussion

This study reveals a close interaction between individual, family and community influences on Jordanian women’s screening behavior. The first theme was seen in the hindering effect of women’s prioritizing children and family needs and in the facilitating effect of their experiencing family and social support to overcome their ambivalence towards prioritizing own health. Women perceived that their main role is to take care of the family. This is consistent with the findings by Trigoni et al.. (2008), who conducted in-depth interviews with 30 women aged 45–65 years in Crete and found that family obligations were one of the reasons for their deferred mammography screening behaviour [27]. Furthermore, Lamyian et al. (2007) interviewed 31 Iranian women and found that the women caring for own health was motivated by their role as caregivers for their households. However, the same study found that competing priorities such as taking time to care for the family was a barrier to Iranian women’s attendance for breast cancer screening [28].

This study showed that women received encouragement to prioritize their own health from husband, family, friends and neighbours. This is consistent with Wagle and co-workers’ (1997) findings about the influence of the social support network in soothing the stress related to cancer and enhancing women’s practice of BSE. Moreover, they suggested that breast health awareness campaigns that address the woman’s formal and informal social support network can positively influence her screening behaviour [29].

On the other hand, women in our study told that some husband’s had misconceptions about breast cancer being a transmissible illness. Men’s knowledge about breast cancer and their attitudes towards their partner’s breast cancer screening is context sensitive and largely unexplored in literature. In their qualitative study Flores and Mata (1995) found that Latino males lacked specific knowledge about their spouse’s breast and cervical cancer screening, procedures, or recommended frequency of such examinations [30]. They suggested that preventive health measures could be improved by a better understanding of the husbands knowledge base and attitudes towards the wife‘s health and health seeking efforts. Conversely, in a postal survey conducted by Chamot and Perneger (2002) in Geneva, men were found as knowledgeable about breast cancer and mammography screening as women but had more favorable attitudes toward breast cancer screening than women [31].

Women in this study perceived cancer as an incurable disease associated with suffering and death, risk of diminished femininity, husband’s rejection, and social stigma. Fear of diminished femininity and treatment suffering was also described by Remennick (2006), social stigma associated with breast cancer was described Baron-Epel et al. (2004) to be attached to those inflicted by the illness and those who go for screening [32,33]. The perceived link between cancer and death was reported by Bener et al. (2001) when he conducted a survey with 1750 Arabic women in the United Arab Emirates [34].

In our study fear can be interpreted as a potential barrier to screening behaviour. Women feared that if they seek screening they might get a breast cancer diagnosis and felt it is better not to know*.* This was also reported by Bener et al. (2002) in his qualitative FGDs study with Arabic women in the United Arab Emirates [35]. This is also consistent with the findings of Petro-Nustas (2001) who assessed the beliefs of a convenience sample of 59 young Jordanian women aged 18 to 45 years towards mammography screening. The study showed that even though 76% of the participants agreed about the benefits of mammography, half of them identified fear of discovering breast cancer as the main barrier to mammography [36]. Fear is often based on lack of breast health knowledge. Our previous study showed that Jordanian women with higher levels of breast health knowledge had significantly more breast health practices compared to those with less knowledge [37].

Our findings showed that women preferred to have their CBE done by a female health provider. This is consistent with previous literature; Ahmad et al. (2001) found that physicians’ gender plays a role in sex-sensitive examination, such as Pap tests and CBE. The study also recommended enhancing physician-patient interactions for sex-sensitive cancer screening examinations by health education initiatives targeting male physicians and women themselves [38]. Another study by Lurie et al. (1993) showed that women are more likely to undergo screening with Pap smears and mammograms if they see female rather than male physicians, particularly if the physician is an internist or family practitioner [39]. In this study women told that their fears towards mammography screening were confirmed by their health care providers. A study by Leslie et al. (2003) showed that health education given to women by their health providers is effective in increasing their knowledge about breast cancer and the benefits of screening [40].

Our finding that the women felt safe and out of the risk zone for breast cancer could be interpreted as a barrier to Jordanian women’s breast health-seeking behaviour. This is consistent with the constructs of the Revised Health Belief Model, as perceived seriousness of and susceptibility to breast cancer influence perceived threat. Similarly, perceived benefits from early detection of breast cancer and perceived barriers to screening influence breast health-seeking behaviour. In addition, general health motivation, self-efficacy and confidence in their ability to successfully perform the behaviour enhance breast health practices [41].

In contrast, we also found that acquiring knowledge and skills made the women feel safe and encouraged them to practice breast health examinations. Juon et al (2004) found that the strongest correlate with regular mammograms was the knowledge of screening guidelines [42]. Secginli and Nahcivan (2006) examined the variables related to the breast cancer screening behaviors of 656 Turkish women and found that knowledge of breast cancer screening guidelines was a major predictor of regular screening [43]. Women in this study told that they received their knowledge about breast cancer and breast health from TV, maternity and child health care female doctors, family members, neighbours, newspapers, radio, internet, magazines, home visits by an outreach social worker and lectures, Previous studies in Jordan suggested that creating awareness through the media and culturally appropriate educational interventions could improve women’s knowledge about breast cancer and early detection examinations [36,37,44,45].

The findings of this study are consistent with previous studies in the Middle East [28,33-37,46-52]. Several potential barriers were reported in the literature to negatively influence Middle Eastern women’s breast health seeking behaviour, including lack of breast health knowledge, lack of physician’s recommendation, fear of cancer, worry about finding a breast tumour, fear of stigma, embarrassment, preference of female health providers, opposition of the husband or other male family members, lack of perceived benefits, perceptions that breast cancer is fatal and not curable, lack of time and lack of accessibility to breast health services [33-37,46-52]. As for religion it was found that it acts as a facilitator in terms of motivating women to take charge of their own health [47] and as a barrier when breast cancer is passively accepted as a test from God [35,48].

We expect this work to enrich the literature by providing a better understanding of the Jordanian women’s ambivalence towards breast cancer and breast health. Moreover, breast health practices are influenced by the socio-cultural context [35,46] and the findings of this study will be used by the JBCP to design breast health promotion interventions that are culturally appropriate and specifically tailored to overcome the barriers and catalyse on the facilitators in Jordan. The strength of our study is in its methodology, including: recruitment of a purposively diverse sample that enriched the in-depth exploration of the material from the focus groups; the rigour of coding; the latent thematic development; and the triangulation of researchers. Still, the findings of this study cannot be generalized to all Jordanian or Arabic women.

## Conclusions

Our findings contribute to a better understanding of Jordanian women’s views of breast cancer and their breast health-seeking behaviour. Breast health awareness interventions need to address women’s fears from breast cancer through emphasizing the good prognosis of the disease when detected early and involving breast cancer survivors to provide a living example of winning the survival battle against breast cancer. Women’s ambivalence in prioritizing own health, their fear of diminished femininity and husband’s rejection could be changed positively through mobilizing family and social support to encourage women to seek early detection of breast cancer.

This study also exposed misconceptions among husbands about breast cancer being contagious and misapprehensions among physicians towards mammography screening. As well there were barriers to women’s accessing breast health care due to lack of female physicians. These constrains should be handled to enhance Jordanian women access to breast screening. Recognizing the voices of Jordanian women could contribute to earlier detection of breast cancer and thus to higher survival rates.

## Competing interests

The authors declare that they have no competing interests.

## Authors' contributions

HT, RQ and RW conceived the study, HT performed data collection, analysis, and drafted the manuscript. VB, RQ, RW and LN participated in the data analysis and adjusted the manuscript. All the authors read and approved the final manuscript.

## Pre-publication history

The pre-publication history for this paper can be accessed here:

http://www.biomedcentral.com/1472-6874/12/21/prepub
